# Biophysical Characterization of LTX-315 Anticancer Peptide Interactions with Model Membrane Platforms: Effect of Membrane Surface Charge

**DOI:** 10.3390/ijms231810558

**Published:** 2022-09-12

**Authors:** Dong Jun Koo, Tun Naw Sut, Sue Woon Tan, Bo Kyeong Yoon, Joshua A. Jackman

**Affiliations:** 1 School of Chemical Engineering and Translational Nanobioscience Research Center, Sungkyunkwan University, Suwon 16419, Korea; 2 School of Healthcare and Biomedical Engineering, Chonnam National University, Yeosu 59626, Korea

**Keywords:** anticancer peptide, oncolytic, peptide, LTX-315, membrane-peptide interactions, quartz crystal microbalance-dissipation, electrochemical impedance spectroscopy

## Abstract

LTX-315 is a clinical-stage, anticancer peptide therapeutic that disrupts cancer cell membranes. Existing mechanistic knowledge about LTX-315 has been obtained from cell-based biological assays, and there is an outstanding need to directly characterize the corresponding membrane-peptide interactions from a biophysical perspective. Herein, we investigated the membrane-disruptive properties of the LTX-315 peptide using three cell-membrane-mimicking membrane platforms on solid supports, namely the supported lipid bilayer, intact vesicle adlayer, and tethered lipid bilayer, in combination with quartz crystal microbalance-dissipation (QCM-D) and electrochemical impedance spectroscopy (EIS) measurements. The results showed that the cationic LTX-315 peptide selectively disrupted negatively charged phospholipid membranes to a greater extent than zwitterionic or positively charged phospholipid membranes, whereby electrostatic interactions were the main factor to influence peptide attachment and membrane curvature was a secondary factor. Of note, the EIS measurements showed that the LTX-315 peptide extensively and irreversibly permeabilized negatively charged, tethered lipid bilayers that contained high phosphatidylserine lipid levels representative of the outer leaflet of cancer cell membranes, while circular dichroism (CD) spectroscopy experiments indicated that the LTX-315 peptide was structureless and the corresponding membrane-disruptive interactions did not involve peptide conformational changes. Dynamic light scattering (DLS) measurements further verified that the LTX-315 peptide selectively caused irreversible disruption of negatively charged lipid vesicles. Together, our findings demonstrate that the LTX-315 peptide preferentially disrupts negatively charged phospholipid membranes in an irreversible manner, which reinforces its potential as an emerging cancer immunotherapy and offers a biophysical framework to guide future peptide engineering efforts.

## 1. Introduction

Cancer is one of the leading causes of human morbidity and mortality [1], and various treatment strategies involving surgical removal, radiotherapy, and chemotherapy are widely used [2]. However, conventional treatments often face challenges such as low selectivity, high cost, side effects, and multidrug resistance [1,3,4,5,6]. To overcome these limitations, emerging cancer treatment strategies are transitioning to immunotherapies, which seek to potentiate the immune system [7,8]. More specifically, cancer immunotherapies use pharmacological agents such as antibodies, small molecules, and peptides to activate specific components of the immune system or inhibit signals that suppress the immune response generated by cancer cells [7,9]. Among the various targeting agents, anticancer peptides (ACPs) have recently emerged as one of the most promising classes of agents due to their high selectivity for cancer cells [10,11], low risk of drug resistance [6], and ability to be chemically modified to improve targeting [12]. Structurally, ACPs are classified as a series of relatively short peptides that consist of around 5–50 amino acids and can inhibit cancer cell proliferation and metastasis and/or prevent tumor vascular formation, i.e., angiogenesis [5]. Some ACPs are synthetic while others are derived from and optimized based on the amino acid sequences of naturally occurring antimicrobial peptides (AMPs) [9,11].

To date, numerous ACPs have been reported to exhibit anticancer activity, and each ACP has a distinct amino acid sequence and secondary structure [11]. At the same time, there are some structural and functional characteristics that ACPs share. These hallmark characteristics include their cationic nature resulting from cationic amino acids (Lys and Arg residues), a large fraction (typically ≥ 50%) of hydrophobic amino acids (Val, Leu, Ile, Phe, and Trp residues) [2,13], and α-helical or β-sheet structures [4,14]. The high selectivity of ACPs originates from the physicochemical and structural properties of cancer cells that are distinct from those of normal cells [2,13]. In particular, cancer cell membranes typically have a greater negative surface charge than that of normal cells due to containing a large fraction of anionic, phosphatidylserine (PS) lipids on the outer leaflet (by contrast, PS lipids are normally restricted to the inner leaflet of non-cancer cells) [15], which enables positively charged ACPs to preferentially bind to them [2,4,6,16]. Additionally, the hydrophobic amino acids of ACPs facilitate penetration into the lipid bilayer structure of cancer cell membranes [17,18].

**Figure 1:**
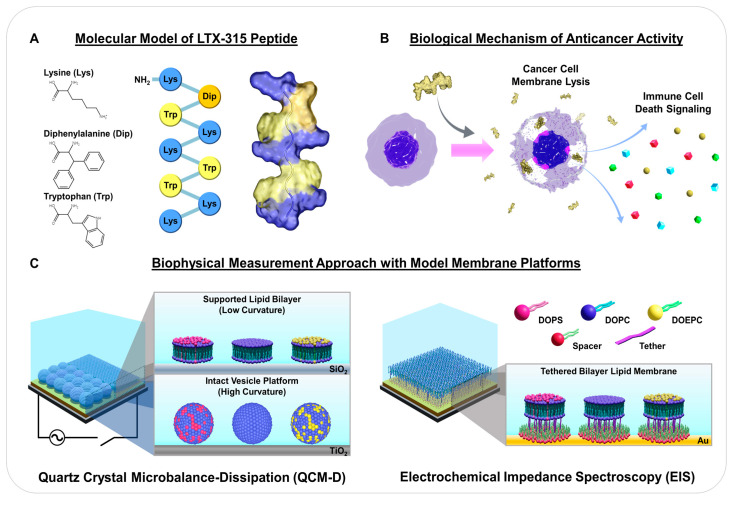
Overview of LTX-315 anticancer peptide and experimental strategy: (**A**) amino acid structures, sequence, and 3D molecular model of LTX-315 peptide. Hydrophobic (Trp and Dip) and cationic (Lys) amino acids are depicted in yellow and blue, respectively; (**B**) proposed biological mechanism of how LTX-315 peptide exhibits anticancer activity based on cancer cell membrane disruption; and (**C**) experimental strategy to track membrane-peptide interactions using the supported lipid bilayer (low curvature), intact vesicle (high curvature), and tethered bilayer lipid membrane platforms with different membrane surface charges. Measurements were conducted using the quartz crystal microbalance-dissipation (QCM-D) and electrochemical impedance spectroscopy (EIS) techniques.

There have been ongoing efforts to modify ACP sequences and/or secondary structures in order to tune physicochemical properties and thus achieve desired pharmaceutical and pharmacokinetic properties [19,20]. In the course of these activities, structural parameters important for anticancer activity have been identified, leading to the discovery of ACPs with improved inhibitory properties based on structure-activity relationship studies [21,22]. For example, LTX-315 (K-K-W-W-K-K-W-Dip-K-NH_2_) is a promising synthetic ACP that is derived from the antimicrobial bovine lactoferricin protein [22,23]. Among several tested versions, LTX-315 exhibited optimized anticancer activity based on a series of chemical modifications, including shortening the overall length and inserting 3,3-diphenylalanine (Dip), which is a bulky, noncoded (non-natural) hydrophobic amino acid [7,21,24] (Figure 1A). Through numerous studies using experimental preclinical animal models, intratumorally injected LTX-315 was shown to be a potential first-in-class oncolytic peptide that is currently in human clinical trials for treating various types of cancer [7].

The latest mechanistic picture is that the LTX-315 peptide can directly permeabilize the plasma membrane of cancer cells to cause tumor necrosis while also interfering with mitochondrial membranes and inducing the release of danger-associated molecular pattern (DAMP) molecules that results in immune cell homing to the tumor region [21,22,23,25,26,27] (Figure 1B). Indeed, DAMP release from permeabilized cancer cells can trigger immunogenic cell death, which is a way in which ACPs such as LTX-315 boost antitumor immunity through various mechanisms (e.g., recruitment of antigen-presenting cells and subsequent tumor-related material uptake) [28]. Such immune-stimulating activities of ACPs in tumor regions are distinct from the broader set of immunomodulatory properties of AMPs, which not only kill microbes but can also act as chemokines and regulate production of other chemokines, inhibit pro-inflammatory cytokine production, and modulate cell responses of the adaptive immune response depending on the specific AMP [29]. Interestingly, while membrane-peptide interactions are the principal driver of the LTX-315 peptide’s mechanism of action, there is an outstanding need to directly characterize the membrane-disruptive properties of the LTX-315 peptide, especially considering how biophysical measurement strategies have helped to advance knowledge about antimicrobial and antiviral peptides [30,31,32,33]. Until now, mechanistic understanding about the LTX-315 peptide has been obtained from cell-based biological experiments and the use of biophysical measurement strategies based on cell-membrane-mimicking model membrane platforms and surface-sensitive measurement approaches would be advantageous.

Towards this goal, herein, we investigated the membrane-disruptive properties of the LTX-315 peptide using three types of model membranes, namely the supported lipid bilayer (SLB), intact vesicle, and tethered bilayer lipid membrane (tBLM) platforms (Figure 1C). Since membrane surface charge is a generally important factor in conferring ACP selectivity, particular focus was placed on evaluating the effects of membrane surface charge on membrane-peptide interactions by utilizing positively charged, neutral, and negatively charged lipid bilayers. Zwitterionic phosphatidylcholine (PC) lipids consisting of 1,2-dioleoyl-*sn*-glycero-3-phosphocholine (DOPC) were used to prepare neutral lipid bilayers that bear resemblance to the main lipid component of normal cell membranes, while anionic PS lipids consisting of 1,2-dioleoyl-*sn*-glycero-3-phospho-L-serine (DOPS) were mixed with PC lipids to fabricate negatively charged lipid bilayers since cancer cell membranes distinctly contain an abundance of PS lipids in the outer leaflet [15]. As a control, cationic ethylphosphatidylcholine (EPC) lipids consisting of 1,2-dioleoyl-*sn*-glycero-3-ethylphosphocholine (DOEPC) were mixed with PC lipids to prepare positively charged lipid bilayers since EPC lipids are ethylated derivatives of PC lipids. The SLB and intact vesicle platforms also exhibit relatively low and high degrees of membrane curvature, respectively, which was a secondary factor considered in the biophysical analyses. All measurements involving the SLB and intact vesicle platforms were conducted using the quartz crystal microbalance-dissipation (QCM-D) technique, which is widely used to track membrane-peptide interactions at solid-liquid interfaces in a label-free format and its time-resolved resonance frequency (Δf) and energy dissipation (ΔD) signals are sensitive to the acoustic mass and viscoelastic properties of the model membrane adlayers, respectively [34,35,36,37]. Additionally, tBLM platform experiments were conducted using the electrochemical impedance spectroscopy (EIS) technique, which tracks membrane-peptide interactions by evaluating changes in the electrical conductance (G_m_) and capacitance (C_m_) properties of the lipid bilayer membrane [38,39,40,41].

## 2. Results and Discussion

While membrane-disruptive AMPs have been widely investigated using biophysical measurement strategies, the LTX-315 anticancer peptide has only been studied from a biological viewpoint so far. Incorporating a biophysical perspective can improve mechanistic understanding about how the LTX-315 peptide works, which led us to begin by characterizing the charge-dependent interactions of the LTX-315 peptide with solution-phase lipid vesicles. The LTX-315 peptide had previously been suggested to exhibit an amphipathic, α-helical structure based on computational predictions [7,22,42] while our circular dichroism spectroscopy experiments indicated that solution-phase LTX-315 peptide is structureless in aqueous conditions and also upon the addition of 50% *v*/*v* trifluoroethanol (TFE) or lipid vesicles (Figure S1). This finding is consistent with its 9-mer amino acid sequence as short peptides are often structureless due to rapid conformational flickering [43]. Furthermore, the LTX-315 peptide exhibited good aqueous solubility, which is in line with its high proportion of cationic amino acids [2,44].

**Figure 2:**
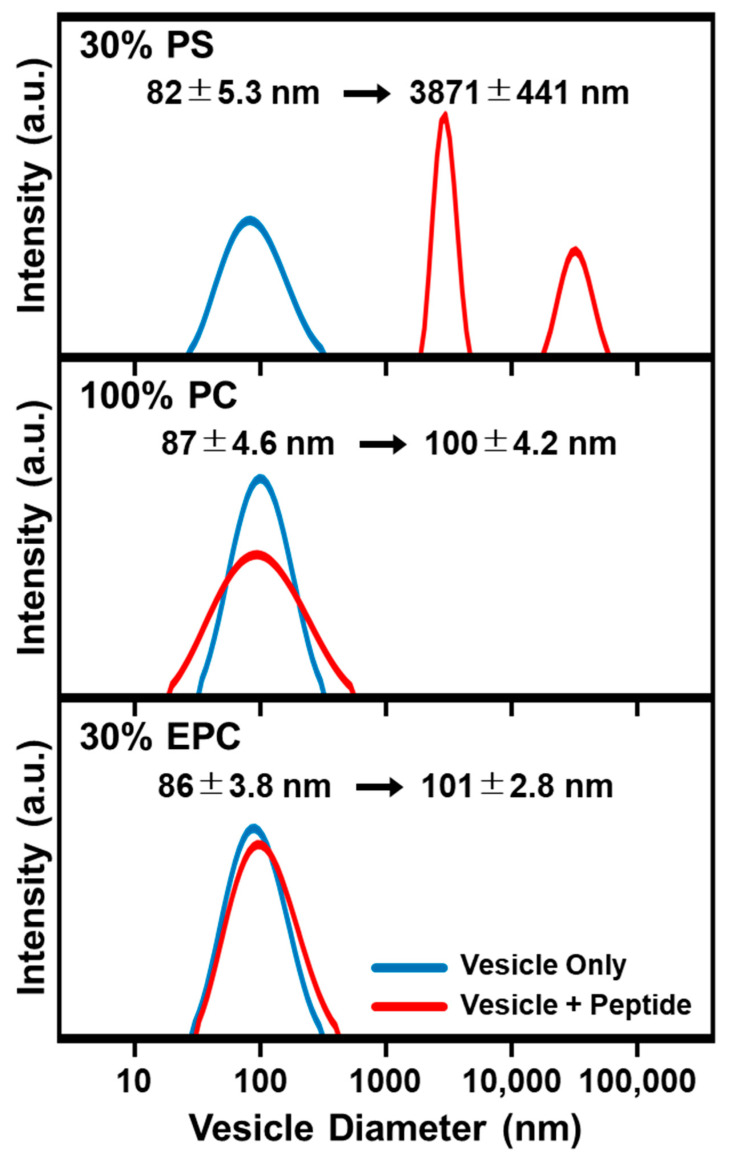
DLS characterization of LTX-315 peptide effects on suspended lipid vesicles with different membrane surface charges. The size distribution of solution-phase lipid vesicles was obtained before (blue lines) and after incubating lipid vesicles with LTX-315 peptide (red lines) by dynamic light scattering (DLS) measurements. Corresponding changes in the size distribution are presented as Gaussian profiles for 70/30 DOPC/DOPS (**top**), 100 DOPC (**middle**), and 70/30 DOPC/DOEPC (**bottom**) lipid vesicles.

We proceeded to incubate 20 μM LTX-315 peptide with suspended, different-charge lipid vesicles of ~85-nm diameter and observed the corresponding effects on solution-phase vesicle size distribution by dynamic light scattering (DLS) measurements (Figure 2). After 30 min incubation, the size distribution of peptide-treated vesicles was checked. LTX-315 peptide had minimal effect on the size distribution of positively charged 70/30 mol% DOPC/DOEPC and zwitterionic 100 mol% DOPC lipid vesicles, whereas peptide treatment caused extensive disruption and aggregation of negatively charged 70/30 mol% DOPC/DOPS lipid vesicles. In the latter case, the mean vesicle diameter increased from ~85 nm to ~3500 nm, which indicates strong and irreversible membrane-disruptive interactions. These findings support that the LTX-315 peptide exhibits charge-dependent membrane interactions and led us to conduct QCM-D experiments to track membrane-peptide interaction kinetics.

The QCM-D experiments were conducted to investigate how LTX-315 disrupts an adsorbed layer of intact vesicles on a titania-coated sensor surface and an SLB platform on a silica-coated sensor surface. In both cases, QCM-D monitoring was employed to track model membrane platform fabrication, followed by LTX-315 peptide addition and subsequent membrane-peptide interactions. In the QCM-D experiments described below, the measurement signals were reset to zero after platform fabrication so that the baseline values at the initial time point correspond to an already fabricated model membrane platform and the main focus was measuring QCM-D Δf and ΔD shifts due to membrane-peptide interactions.

### 2.1. Intact Vesicle Platform

**Figure 3:**
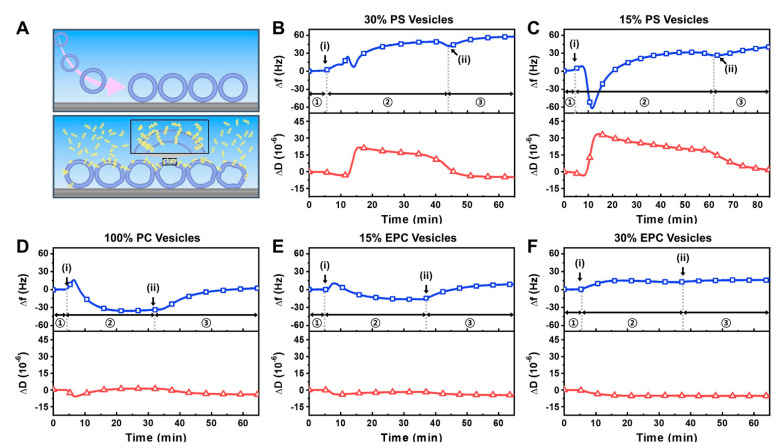
QCM-D tracking of LTX-315 peptide interactions with intact vesicle adlayer depending on membrane surface charge: (**A**) schematic illustration of intact vesicle adlayer on TiO_2_-coated sensor surface before and after peptide addition; (**B**–**F**) corresponding QCM-D measurement kinetics for peptide addition to (**B**) 70/30 DOPC/DOPS, (**C**) 85/15 DOPC/DOPS, (**D**) 100 DOPC, (**E**) 85/15 DOPC/DOEPC; and (**F**) 70/30 DOPC/DOEPC lipid vesicle adlayers. In each panel, the QCM-D Δf (top, blue squares) and ΔD (bottom, red triangles) shifts are presented as a function of time and the initial baseline signals correspond to the intact vesicle adlayer. Stages 1, 2, and 3 correspond to intact vesicle platform alone, during peptide addition, and during buffer washing, respectively. Arrows i and ii denote peptide addition and buffer washing steps, respectively.

We investigated the interaction of the LTX-315 peptide with intact vesicle adlayers possessing different membrane surface charges (Figure 3A). Negatively charged vesicles were composed of 70 or 85 mol% zwitterionic DOPC lipid and 30 or 15 mol% anionic DOPS lipid, respectively, while zwitterionic vesicles were composed of 100 mol% DOPC lipid only. On the other hand, positively charged vesicles were composed of 70 or 85 mol% zwitterionic DOPC lipid and 30 or 15 mol% cationic DOEPC lipid, respectively. With increasing DOPS or DOEPC lipid fraction, the vesicles had greater charge magnitude. In all cases, the ~80-nm diameter, extruded vesicles adsorbed onto titania-coated sensor surfaces and remained intact, resulting in close-packed vesicle adlayers with QCM-D shifts that are consistent with literature values and composition-dependent trends (Figure S2) [31,45,46,47]. After intact vesicle adlayer formation, we proceeded to conduct a buffer washing step and then added 20 µM LTX-315 peptide to the different vesicle adlayers.

For negatively charged 70/30 mol% DOPC/DOPS vesicles, the Δf signal increased immediately by around 45 Hz upon peptide addition and showed complex, multi-step interaction kinetics that are indicative of membrane disruption (Figure 3B) [48,49,50]. The rapid increase in the Δf signal is reminiscent of when certain antimicrobial and viral-derived peptides disrupt intact vesicle adlayers via transmembrane insertion [51,52]. The corresponding ΔD signal also rapidly increased by around 20 × 10^−6^ due to the initial membrane-peptide interaction, which further supports extensive membrane disruption and the ΔD signal gradually decreased but remained high. Upon a buffer washing step to remove free peptide from the measurement chamber, the Δf signal stayed high and pointed to the loss of vesicle adlayer mass from the sensor surface while the ΔD signal decreased back to around the baseline value prior to peptide addition.

Similarly, for negatively charged 85/15 mol% DOPC/DOPS vesicles, LTX-315 peptide caused extensive membrane disruption, however, the interaction kinetics were distinct and showed an initially rapid and sharp drop in the Δf signal by around −60 Hz, followed by an increase back to around 40 Hz relative to the vesicle baseline (Figure 3C). Such interaction kinetics are often seen with antimicrobial peptides that exhibit a carpet-like mechanism, whereby peptides adsorb onto the vesicle surface, causing the initial Δf shift decrease due to peptide binding and vesicle swelling followed by a subsequent Δf shift increase due to extensive membrane disruption after a critical surface density of bound peptide is reached [51,53,54]. Interestingly, the corresponding ΔD signal in this case rapidly increased by around 35 × 10^−6^ before gradually decreasing to around 25 × 10^−6^. Upon buffer washing, the Δf signal again stayed high and the ΔD signal decreased to around 1 × 10^−6^ relative to the vesicle baseline. Together, these findings support that the LTX-315 peptide causes extensive membrane disruption of DOPS-containing lipid vesicle compositions and the specific membrane interaction profile depended on the DOPS lipid fraction whereby a larger DOPS fraction caused more immediate disruption.

In marked contrast, the addition of LTX-315 peptide to 100 mol% DOPC lipid vesicles appeared to mainly involve peptide binding only, as indicated by a Δf shift of around −30 Hz and a nearly negligible ΔD shift (Figure 3D). Upon buffer washing, the Δf shift increased to around 3 Hz relative to the vesicle baseline, supporting that peptide binding to the vesicles was modest while the negligible ΔD shift further indicated that peptide binding was the main interaction event rather than peptide-induced membrane disruption. A similar membrane interaction profile was observed when LTX-315 peptide was added to positively charged 85/15 mol% DOPC/DOEPC lipid vesicles while there was less bound peptide, as indicated by a Δf shift decrease of only around −12 Hz and a negligible ΔD shift (Figure 3E). Upon buffer washing, nearly all bound peptide was removed, supporting that there was only weak peptide attachment and the final Δf shift was around 10 Hz. On the other hand, for 70/30 mol% DOPC/DOEPC lipid vesicles, there was minimal peptide interaction, as indicated by a final Δf shift of around 14 Hz along with a negligible ΔD shift (Figure 3F). These results support that significant peptide binding can occur to zwitterionic lipid vesicles while minor to negligible peptide binding occurred for lipid vesicles with increasingly positive membrane surface charge.

**Figure 4:**
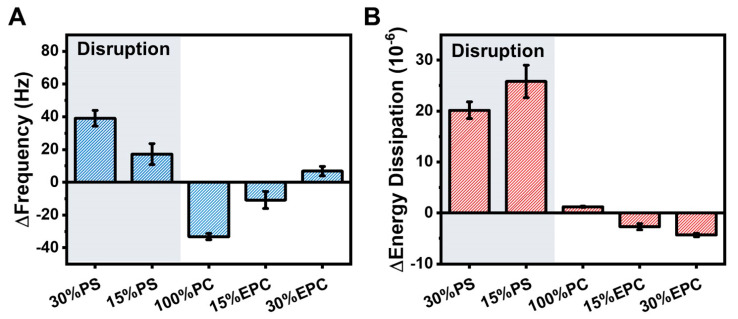
Summary of QCM-D measurement responses for LTX-315 peptide interactions with intact vesicle adlayers. Maximum responses of the (**A**) Δf and (**B**) ΔD shifts are presented based on the data in Figure 3 and reported as the mean ± standard deviation from *n* = 3 measurements.

A summary of the maximum QCM-D responses due to LTX-315 peptide addition is presented in Figure 4 and shows a clear dependence on membrane surface charge. For negatively charged DOPS-containing vesicles, there were large positive Δf shifts of up to around 40 Hz due to peptide-induced membrane disruption along with large ΔD shifts due to membrane structural rearrangements during the interaction process. Notably, greater membrane disruption occurred for more negatively charged lipid vesicles that contained larger DOPS fractions. In some cases, the corresponding ΔD shifts increased by up to 295% relative to the vesicle baseline (Figure S3). Conversely, for zwitterionic DOPC lipid vesicles, peptide binding was the main interaction event and the corresponding Δf shifts were around −33 Hz on average while the ΔD shifts were negligible. Moreover, for positively charged DOEPC-containing lipid vesicles, there were comparatively small Δf shifts along with minor ΔD shifts, which together indicate weak peptide interactions.

Considering that the LTX-315 peptide is positively charged, these composition-dependent results support that peptide-induced membrane disruption of negatively charged lipid vesicles occurs via attractive electrostatic interactions while weaker interactions occur with zwitterionic lipid vesicles, in which case peptide binding is the main interaction event. On the other hand, for positively charged lipid vesicles, there is appreciably less peptide binding with increasingly positive membrane surface charge due to repulsive electrostatic interactions.

### 2.2. Supported Lipid Bilayer Platform

**Figure 5:**
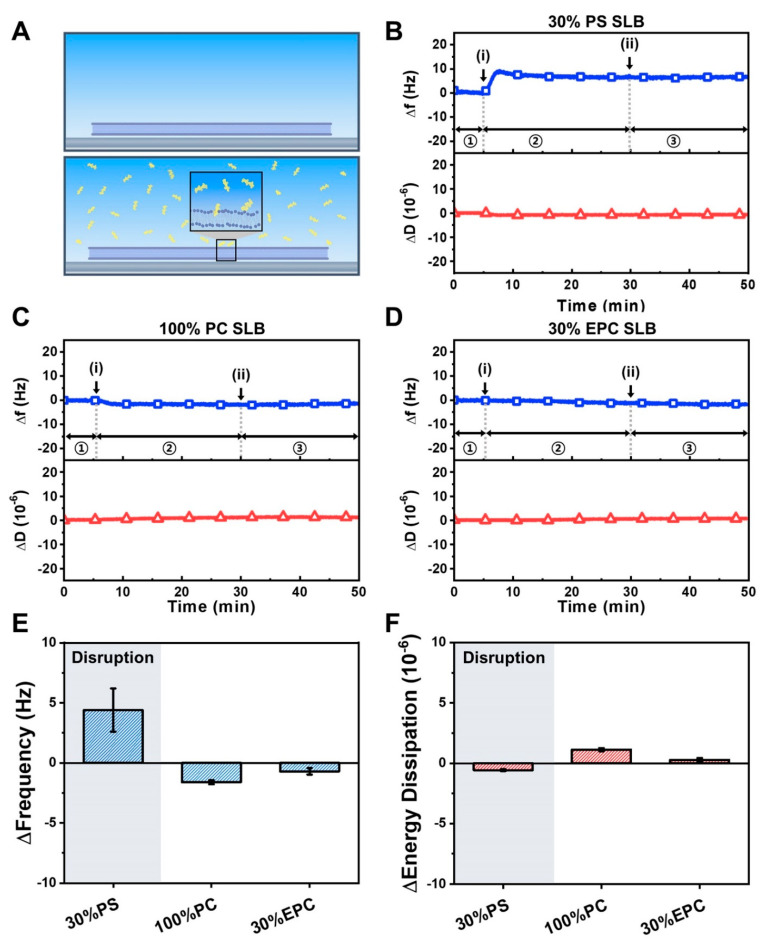
QCM-D tracking of LTX-315 peptide interactions with supported lipid bilayers depending on membrane surface charge: (**A**) schematic illustration of supported lipid bilayer on SiO_2_-coated sensor surface before and after peptide addition; (**B**–**D**) corresponding QCM-D measurement kinetics for peptide addition to (**B**) 70/30 DOPC/DOPS, (**C**) 100 DOPC, and (**D**) 70/30 DOPC/DOEPC supported lipid bilayers. In each panel, the QCM-D Δf (top, blue squares) and ΔD (bottom, red triangles) shifts are presented as a function of time and the initial baseline signals correspond to the supported lipid bilayer platform. Arrows i and ii denote peptide addition and buffer washing steps, respectively; (**E**,**F**) summary of QCM-D measurement responses for LTX-315 peptide interactions with supported lipid bilayers. In panels (**B**–**D**), stages 1, 2, and 3 correspond to supported lipid bilayer platform alone, during peptide addition, and during buffer washing, respectively. Maximum responses of the (**E**) Δf and (**F**) ΔD shifts are presented based on the data in panels (**B**–**D**) and are reported as the mean ± standard deviation from *n* = 3 measurements.

Complementing the intact vesicle experiments, we also investigated the interaction of the LTX-315 peptide with supported lipid bilayer (SLB) platforms possessing different membrane surface charges (Figure 5A). Based on the intact vesicle platform data, we selected three lipid compositions for SLB platform testing, including negatively charged 70/30 mol% DOPC/DOPS, zwitterionic 100 mol% DOPC, and positively charged 70/30 mol% DOPC/DOEPC lipid compositions. The SLB platforms were fabricated on silica-coated sensor surfaces by utilizing the vesicle fusion or solvent-assisted lipid bilayer (SALB) method as appropriate. In all cases, the QCM-D Δf and ΔD shifts were around −26 Hz and <1 × 10^−6^, respectively (Figure S4) [37,55].

When 20 µM LTX-315 peptide was added to the negatively charged 70/30 mol% DOPC/DOPS SLB platform, there was a rapid Δf shift increase of around 5 to 8 Hz that indicated membrane disruption (Figure 5B) [50,56]. Upon buffer washing, the Δf shift response remained while there was no change in the ΔD signal throughout the interaction process. On the other hand, there were negligible LTX-315 peptide interactions with zwitterionic 100 mol% DOPC and positively charged 70/30 mol% DOPC/DOEPC SLB platforms (Figure 5C,D). A summary of the QCM-D Δf and ΔD shifts further supports that the LTX-315 peptide disrupts negatively charged SLBs only whereas it has negligible effects on zwitterionic and positively charged SLBs (Figure 5E,F). These results reinforce that membrane surface charge plays an important role in mediating membrane attachment of the LTX-315 peptide while negligible peptide binding to zwitterionic SLBs—in marked contrast to the moderate binding observed to intact, zwitterionic lipid vesicles—suggests that membrane curvature has a secondary effect.

### 2.3. Tethered Bilayer Lipid Membrane Platform

In addition to the QCM-D measurements, we also investigated how the LTX-315 peptide interacts with tBLM platforms possessing different membrane surface charges by using the EIS technique. Briefly, DOPS or DOEPC lipids were mixed with DOPC lipids in ethanol in desired ratios to prepare negatively or positively charged mobile lipid aliquots. During the tBLM fabrication process, the mobile lipid aliquot was introduced on top of the tethered lipid monolayer to serve as the top layer of the tBLM. After establishing a baseline with the fabricated tBLM platform, 20 µM LTX-315 peptide was added to the tBLM platforms for 30 min, followed by a PBS buffer washing step. The EIS technique tracked changes in the conductance (G_m_) and capacitance (C_m_) signal of the tBLM platform. When a peptide causes membrane disruption, the G_m_ signal increases due to greater ion flow across the more permeable membrane, while an increase in the C_m_ signal indicates membrane thinning of the tethered lipid bilayer, which provides insight into membrane structural integrity [38,57,58]. Furthermore, changes in the frequency and the phase value of phase minima of the EIS spectra were evaluated using Bode plot analysis, which provides insight into the state of ion leakage that corresponds to the G_m_ signal and membrane thinning corresponding to the C_m_ signal, respectively [59].

**Figure 6:**
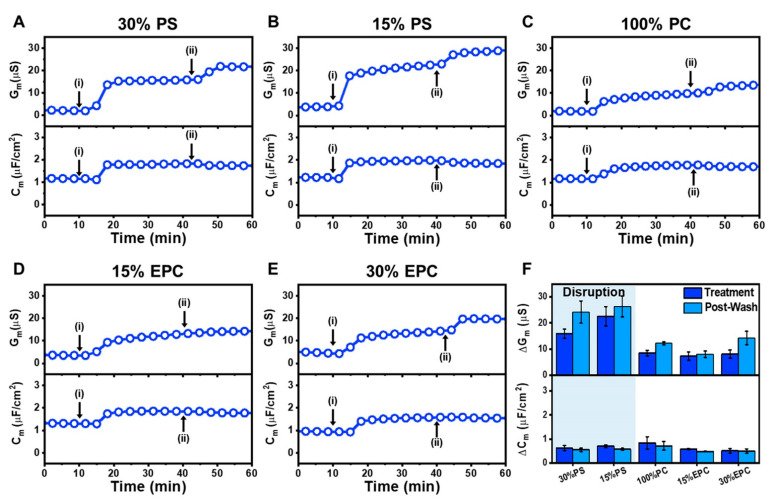
EIS tracking of LTX-315 peptide interactions with tethered lipid bilayers depending on membrane surface charge: (**A**) time-dependent changes in conductance (G_m_) and capacitance (C_m_) signals upon LTX-315 peptide addition to a 70/30 DOPC/DOPS tBLM platform. LTX-315 peptide was added to the tBLM platform starting at *t* = 10 min (arrow i), followed by a buffer washing step from *t* = 30 min onward (arrow ii); corresponding EIS data for (**B**) 85/15 DOPC/DOPS, (**C**) 100 DOPC, (**D**) 85/15 DOPC/DOEPC, and (**E**) 70/30 DOPC/DOEPC tBLM platform; and (**F**) summary of G_m_ and C_m_ shifts upon LTX-315 peptide addition (treatment) and after buffer washing (post-wash) for tBLMs with different membrane surface charges. The data are reported as the mean ± standard deviation from *n* = 3 measurements.

On negatively charged 70/30 mol% DOPC/DOPS tBLMs, the obtained G_m_ and C_m_ baselines were <3.5 µS and ~1.1 µF/cm^2^_,_ respectively. LTX-315 peptide addition led to increases in the G_m_ and C_m_ signals to around 16 µS and 1.8 µF/cm^2^, respectively (Figure 6A). After buffer washing, the G_m_ shift increased to around 22 µS while the C_m_ shift slightly decreased to around 1.7 µF/cm^2^. In the case of 85/15 mol% DOPC/DOPS tBLMs, the measured baselines were <5.5 µS for G_m_ and ~1.2 µF/cm^2^ for C_m_. A similar trend in interaction kinetics occurred whereby the G_m_ and C_m_ signals increased to around 22 µS and 2 µF/cm^2^, respectively (Figure 6B). Following a subsequent rinsing step, the final G_m_ and C_m_ shifts were around 28 µS and 1.8 µF/cm^2^, respectively. On the other hand, starting with G_m_ and C_m_ baselines of <2.5 µS and ~1.2 µF/cm^2^, the addition of LTX-315 peptide to zwitterionic 100 mol% DOPC tBLMs induced G_m_ and C_m_ shifts of around 9 µS and 1.8 µF/cm^2^, respectively, and the corresponding values after buffer washing were around 13 µS and 1.7 µF/cm^2^, respectively (Figure 6C).

On positively charged 85/15 mol% DOPC/DOEPC tBLMs with G_m_ and C_m_ baselines of <3.5 µS and ~1.3 µF/cm^2^, respectively, LTX-315 peptide addition caused corresponding increases in G_m_ and C_m_ values to around 13 µS and 1.8 µF/cm^2^, respectively (Figure 6D). After the following buffer washing step, the recorded G_m_ and C_m_ shifts were around 14 µS and 1.8 µF/cm^2^, respectively. A similar interaction kinetic profile was observed for 70/30 mol% DOPC/DOEPC tBLMs. In this case, the G_m_ and C_m_ baselines were around <5.6 µS and ~1.0 µF/cm^2^, respectively, and the G_m_ and C_m_ signals rose to around 14 µS and 1.6 µF/cm^2^ (Figure 6E). The corresponding values after buffer washing were around 20 µS and 1.5 µF/cm^2^, respectively.

Figure 6F summarizes the EIS shifts in response to LTX-315 peptide addition and to subsequent buffer washing. For 70/30 mol% DOPC/DOPS tBLMs, the addition of LTX-315 peptide caused G_m_ and C_m_ shift increases to around 15.9 ± 1.8 µS and 0.6 ± 0.1 µF/cm^2^, respectively. After washing, the G_m_ shift increased further to 24.1 ± 4.3 µS and the C_m_ shift decreased slightly to 0.6 ± 0.1 µF/cm^2^. While the 85/15 mol% DOPC/DOPS tBLM had less DOPS lipid fraction-wise, LTX-315 peptide addition in this case yielded larger G_m_ and C_m_ shifts of around 22.5 ± 3.7 µS and 0.7 ± 0.1 µF/cm^2^, respectively, which is consistent with the QCM-D data. Subsequent buffer washing caused an increase in the G_m_ shift to around 26.3 ± 4.0 µS and a decrease in the C_m_ shift to around 0.6 ± 0 µF/cm^2^. In the case of zwitterionic 100 mol% DOPC tBLMs, the G_m_ and C_m_ shifts were smaller and around 8.4 ± 1.1 µS and 0.8 ± 0.3 µF/cm^2^, respectively, upon peptide addition, and 12.2 ± 0.6 µS and 0.7 ± 0.2 µF/cm^2^ after buffer washing. Similarly, on 85/15 mol% DOPC/DOEPC tBLMs, LTX-315 peptide addition resulted in G_m_ and C_m_ shifts of 7.3 ± 1.5 µS and 0.6 ± 0 µF/cm^2^, respectively, which slightly changed to around 8.0 ± 1.3 µS and 0.5 ± 0 µF/cm^2^ upon buffer washing. As for 70/30 mol% DOPC/DOEPC tBLMs, the G_m_ and C_m_ shifts were 8.1 ± 1.5 µS and 0.5 ± 0.1 µF/cm^2^, respectively, upon peptide treatment, followed by shift increases to 14.2 ± 2.6 µS and 0.5 ± 0.1 µF/cm^2^ upon buffer washing.

Overall, across all tested lipid compositions, LTX-315 peptide addition and subsequent buffer washing caused increases in both the G_m_ and C_m_ signals, which is consistent with increased membrane permeability and a thinning effect [38]. Notably, the EIS results demonstrated that the G_m_ and C_m_ shifts persisted even after buffer washing, further supporting that peptide-mediated membrane disruption is irreversible. Moreover, the magnitude of the EIS G_m_ shifts strongly depended on the lipid composition. The final G_m_ shifts were around 20–30 µS for negatively charged DOPC/DOPS tBLMs, while the corresponding G_m_ shifts were appreciably smaller around 10–15 µS for zwitterionic DOPC and positively charged DOPC/DOPS tBLMs. Furthermore, Bode plot analysis of the EIS data indicated that the phase minima shifted to higher frequencies and larger phase values upon LTX-315 peptide treatment, and the shifts remained similarly large after buffer washing (Figure S5) [59]. The latter finding additionally supports that LTX-315 peptide causes irreversible membrane disruption, highlighting that the LTX-315 peptide can generally cause tBLM disruption while the extent is appreciably greater for negatively charged membranes.

### 2.4. Mechanistic Analysis of Membrane-Peptide Interactions

**Figure 7:**
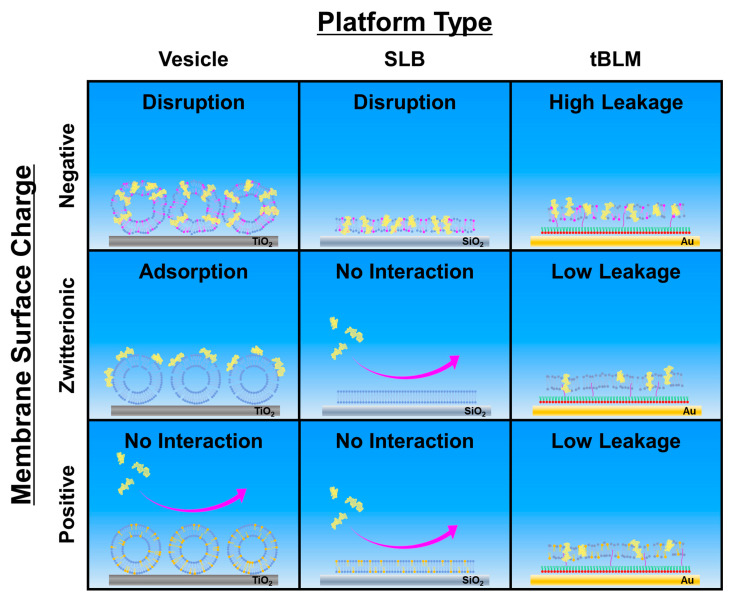
Schematic summary of LTX-315 peptide interactions with lipid membranes and relevant mechanistic factors. The two main tested parameters were membrane surface charge and nanoarchitecture. In general, LTX-315 preferentially disrupts negatively charged membranes and demonstrates enhanced attachment to curved membranes over planar membranes. It should be noted that peptide attachment is a necessary but insufficient step for triggering membrane disruption, which was strongly related to the lipid composition.

A schematic summary of the different membrane interaction outcomes is presented in Figure 7 and illustrates how the membrane-disruptive properties of LTX-315 depend on both membrane surface charge and curvature. For intact vesicle adlayers, LTX-315 can be broadly categorized as: (1) disrupting negatively charged membranes; (2) binding to but not disrupting zwitterionic membranes; and (3) having relatively negligible interactions with positively charged membranes. Notably, more extensive membrane disruption was observed for membranes with larger anionic lipid fractions based on the final QCM-D Δf shifts. For SLB platforms, the peptide only demonstrated attachment to and disruption of negatively charged membranes but neither attached to nor disrupted zwitterionic or positively charged membranes.

These biophysical findings support that LTX-315 peptide preferentially disrupts negatively charged membranes while it is also interesting that peptide molecules could attach to zwitterionic and positively charged lipid vesicles to some extent, whereas negligible binding occurred to SLBs of the same compositions. This latter distinction suggests that membrane curvature influences initial peptide attachment, which has been discussed for other amphipathic lipids and peptides [60,61], and might relate to curvature-induced defects in lipid packing that can facilitate initial peptide attachment in this case.

On the other hand, for tBLM platforms, the LTX-315 peptide also demonstrated extensive membrane disruption of negatively charged membranes while a moderate degree of membrane disruption was also observed for zwitterionic and positively charged membranes. However, it should be noted that the magnitudes of the membrane-disruptive effects in all cases were still appreciably smaller than those caused by more indiscriminate, membrane-solubilizing surfactants, underscoring that the membrane-disruptive effects of LTX-315 peptide are more discriminate and possibly related to a carpet-type mechanism [62]. While the SLB platform is rigidly attached to silica surfaces, the tBLM platform has a low tether density on gold surfaces, which enables greater membrane flexibility to accommodate more peptide binding and hence membrane-peptide interactions are more favorable in general [63]. Nevertheless, the findings across the three model membrane platforms support that the LTX-315 peptide preferentially disrupts negatively charged membranes, which also agrees well with the DLS results.

The membrane-disruptive effects of the LTX-315 peptide to inhibit PS-enriched lipid membranes mimicking cancer cell membrane compositions is noteworthy due to its combination of preferential targeting of negatively charged membranes (i.e., selectivity) and irreversible membrane damage. For example, the appreciably longer, 37-mer human cathelicidin AMP, LL-37, also has anticancer activity and induces irreversible membrane disruption [38,64], however, its membrane-disruptive interactions occur largely independently of membrane surface charge and are hence not selective [65,66]. Likewise, the short, cationic AMP, Aurein 1.2, has been reported to cause irreversible disruption of tBLM platforms, but its membrane disruption is not charge-dependent [67]. Similar trends, i.e., irreversible disruption but no charge selectivity, have also been reported for cationic, small molecules that mimic short AMPs [68].

In addition, other cationic AMPs such as PGLa (peptide antibiotic found on frog skin), melimine (a chimera of the melittin and protamine antimicrobial peptides), and cys-melimine (melimine with an additional cysteine on the N-terminus) have been found to exhibit selectivity for negatively charged membranes but could only induce transient and reversible membrane disruption of tBLM platforms mimicking bacterial cell membrane compositions [39,41]. There are also various other short, cationic peptides that exhibit cell-penetrating, antimicrobial, and/or anticancer properties [69], some of which can be briefly covered here to compare with LTX-315. For example, the cell-penetrating TAT (48–60) peptide does not exhibit selectivity to negatively charged membranes [70], while the cell-penetrating pep-1 peptide exhibits a modest degree of charge selectivity and membrane disruption [71] (see also evidence of pep-1 and an engineered derivative causing only weak membrane disruption and negligible or reversible vesicle aggregation; Ref. [72]). Recently, a series of short, 13-mer cationic ACPs with α-helical character have been reported to exhibit selective inhibition of cancer cells in vitro, and demonstrated stronger interactions with negatively charged lipid monolayers vs. neutral lipid monolayers at the air/water interface, which was correlated with the degree of induced α-helicity due to membrane partitioning [73]. It has also been reported that peptide conformational flexibility is a key factor related to the anticancer activity of other cationic, α-helical peptides [74], and has been noted for cationic, β-sheet peptides as well [75]. Interestingly, the 9-mer LTX-315 peptide is structureless in both aqueous solution and in membrane environments according to our CD spectroscopy results and hence its membrane-disruptive activity does not depend on peptide conformational changes, which suggests that LTX-315 potentially fits within a distinct class of ACPs from a structure-function perspective.

From a translational viewpoint, our findings provide biophysical insight into possibly why the LTX-315 peptide selectively inhibits cancer cells over normal cells, as indicated by greater inhibitory potency towards cancer cells vs. normal cells that has been observed in in vitro testing [22]. Indeed, it has been suggested that this selectivity to inhibit cancer cells may relate to differences in the physicochemical properties of cancer and normal cells [7,23,27]. While anionic PS lipids are mainly located on the inner leaflet of normal cell membranes, they are present on the outer surface of cancer cell membranes, which is a prominent distinction that results in cancer cell membranes typically having greater negative surface charge than normal cell membranes and also helps cancer cells avoid being recognized as threats by the immune system [76,77].

Returning to our findings, these compositional features of cancer and normal cell membrane surfaces help to explain why LTX-315 preferentially disrupts cancer cell membranes due to strong electrostatic interactions and is consistent with the biophysical results observed in this study across the three tested model membrane platforms. Conversely, the outer leaflet of normal cell membranes contains high zwitterionic lipid fractions, especially PC lipids [78], and hence the LTX-315 peptide is more likely to interact only weakly with those membranes and not cause such intense membrane disruption, which also agrees well with our biophysical results. It should be noted that the model membrane platforms in this study were designed to mimic this key compositional difference in cell membrane properties (i.e., PS content level reflecting the outer leaflet composition of cancer vs. normal cell membranes) and, while cancer cell membranes are inherently more structurally complex than model membranes, our findings are consistent with the aforementioned in vitro cell inhibition results [22] and suggest that the biophysical measurement approach utilized herein might be useful to evaluate the membrane-disruptive properties of ACP candidates, with LTX-315 serving as a benchmark to guide future peptide engineering efforts as well as to test other ACPs with distinct secondary structures and conformational properties. Collectively, these findings also provide a biophysical basis to reinforce that the LTX-315 peptide is a promising ACP because it exhibits irreversible membrane-disruptive properties, which are related to a compositional feature that is preferentially found on the outer surface of cancer cell membranes and hence can potentially be utilized for therapeutic applications.

## 3. Materials and Methods

### 3.1. Peptide

The LTX-315 peptide (>90% purity) was synthesized by Anygen (Gwangju, Republic of Korea). The amino acid sequence of LTX-315 is Lys-Lys-Trp-Trp-Lys-Lys-Trp-Dip-Lys-NH_2_. The lyophilized peptide was solubilized in deionized, Milli-Q-treated water (MilliporeSigma, Burlington, MA, USA) in order to prepare a stock solution with 20 μM peptide concentration. The molar concentration of peptide in solution was determined by UV-vis absorbance measurements at 280 nm wavelength (i.e., corresponding to maximum absorbance intensity; see Refs. [79,80,81,82]) by using a Boeco-S220 spectrophotometer (Boeco, Hamburg, Germany). Before experiment, an aliquot of the peptide stock solution was diluted with aqueous buffer solution [10 mM Tris buffer (pH 7.5) with 150 mM NaCl]. The amino acid structure and LTX-315 3D molecular model were rendered using the ChemAxon Marvin JS chemical sketcher (https://chemaxon.com/products/marvin-js, accessed on 13 August 2022) and PyMOL 1.3 (Schrödinger, Inc., New York, NY, USA) software packages, respectively.

### 3.2. Vesicle Preparation

1,2-Dioleoyl-*sn*-glycero-3-phosphocholine (DOPC), 1,2-dioleoyl-*sn*-glycero-3-phospho-L-serine (sodium salt) (DOPS), and 1,2-dioleoyl-*sn*-glycero-3-ethylphosphocholine (chloride salt) (DOEPC) lipids dissolved in chloroform were obtained from Avanti Polar Lipids (Alabaster, AL, USA). Small unilamellar vesicles (SUV) were prepared by the extrusion method as follows: dry lipid films were prepared by depositing the appropriate quantity and composition of lipids dispersed in chloroform in a glass vial, evaporating the chloroform solvent with a stream of nitrogen gas at room temperature, and storing the vial in the vacuum state overnight to remove residual chloroform [83]. Then, multilamellar vesicles were generated by hydrating the dry lipid films in an aqueous buffer solution to achieve a bulk lipid concentration of ~5 mg·mL^−1^, and the solution was next subjected to vortexing. Finally, the resulting multilamellar vesicles were extruded through a polycarbonate filter with 50-nm-diameter pores for 31 times in total by using a MiniExtruder (Avanti Polar Lipids). The average diameters of the extruded vesicles were ~80–85 nm, as determined by dynamic light scattering measurements. The vesicle samples for the experiments were prepared by diluting the obtained SUV solution to a 0.1 mg·mL^−1^ lipid concentration. To fabricate intact vesicle and SLB platforms, the SUVs were diluted in 10 mM Tris buffer (pH 7.5) with 150 mM NaCl. The only exception was SLB fabrication for the 70/30 mol% DOPC/DOPS composition, which was formed using the solvent-assisted lipid bilayer (SALB) method as previously described [84,85].

### 3.3. Circular Dichroism (CD) Spectroscopy

CD spectroscopy experiments were performed to characterize the secondary structure of the LTX-315 peptide by using a J-1500 circular dichroism spectrometer (Jasco, Tokyo, Japan) with a 1-mm path length quartz cuvette (Hellma, Müllheim, Germany). For these particular experiments, LTX-315 peptide and vesicle concentration were fixed at 100 μM and 5 mM, respectively, and prepared with 10 mM phosphate buffer [pH 7.5]. In experiments involving vesicles, before the experiment, the peptides and vesicles were mixed and incubated for 30 min. Each spectrum was recorded at 25 °C from 190 to 260 nm using a bandwidth of 1 nm, and the measurements were repeated three times and averaged accordingly. The condition before peptide addition was set as the baseline and subtracted from the data obtained after peptide addition. The averaged spectra values were converted into mean residue molar ellipticity units ([*θ* = *θ*/10 × *c* × *l*]), where *θ* is the ellipticity, *c* is the molar concentration of peptide, and *l* is the path length in cm. The plotted spectra were smoothed by the Savitzky−Golay method (polynomial order: 2). The fractional helicity (fH) of the peptide for each case was calculated as follows:

(1) $$\mathrm{fH}=\left({\left[\theta \right]}_{222}^{Obs}-3000\right)/\left(-36000-3000\right),$$

where ${\left[\theta \right]}_{222}^{\mathrm{Obs}}$ is the molar ellipticity at 222 nm [86,87].

### 3.4. Dynamic Light Scattering (DLS)

DLS measurements were performed to investigate the size distribution of lipid vesicles before and after treatment with LTX-315 peptide. The concentrations of LTX-315 peptide and vesicles were fixed at 20 μM and 1 mM, respectively. The vesicles and peptide were then incubated together for 30 min at room temperature, before the peptide-treated vesicle size distribution was measured. The DLS measurements were conducted using an ELSZ-2000 instrument (Otsuka Electronic Co., Ltd., Osaka, Japan). Vesicle diameters are reported from the intensity-weighed, Gaussian-fitted distribution, as previously described [88].

### 3.5. Quartz crystal Microbalance-Dissipation (QCM-D)

The membrane-disruptive properties of the LTX-315 peptide were characterized by using a Q-Sense E4 instrument (Biolin Scientific AB, Gothenburg, Sweden), as previously described [37]. Silica- and titania-coated QCM-D sensor chips (model nos. QSX303 and QSX310, Biolin Scientific AB) were used for experiments involving SLB and intact vesicle platforms, respectively. Before experiment, the sensor chips were rinsed with 1% (*wt*/*v*) sodium dodecyl sulfate (SDS), water, and ethanol sequentially. After drying with a stream of nitrogen gas, each sensor chip was treated with oxygen plasma for ~1 min by using a CUTE-1MPR machine (Femto Science Inc., Hwaseong, Republic of Korea). Afterwards, the sensor chips were mounted in the measurement chambers and signal baselines were first establishing by injecting buffer solution into the measurement chambers. All liquid samples were introduced by using a Reglo Digital MS-4/6 peristaltic pump (Ismatec, Glattsburg, Switzerland) with a volumetric flow rate of 50 µL·min^−1^. The temperature in each QCM-D measurement chamber was set at 25 °C during experiment, and the resonance frequency (Δf) and energy dissipation (ΔD) shifts were monitored as a function of time. Measurement data were collected at several odd overtones (n = 3–13), and the presented data are reported from the fifth overtone (n = 5) and normalized according to the overtone number.

### 3.6. Electrochemical Impedance Spectroscopy (EIS)

A functionalized gold electrode slide was obtained from SDx Tethered Membranes (Sydney, Australia) and had been precoated with a benzyl-disulfide ethylene glycol monolayer that had a tether-to-spacer molar ratio of 1:9. The sulfur groups of the hydroxyl-terminated benzyldisulphide tetraethylene glycol spacer and the benzyldisulphide polyethylene glycol phytanyl tether covalently attach to the gold electrode surface to form a mixed monolayer [41]. Note that the tether is relatively longer than the spacer, which laterally separates the subsequently formed tethered lipid bilayer from the electrode surface to facilitate an ionic reservoir. This surface functionalization scheme is suitable for preparing tBLM platforms according to the manufacturer’s protocol. Specifically, the gold electrode slide was first rinsed with ethanol and partially dried before being mounted to the tethaPlate measurement chamber (SDx Tethered Membranes) that contained six flow cells (see also ref. [89]). A 3 mM lipid solution in ethanol of the desired lipid composition was prepared and an 8 µL aliquot was introduced into each flow cell, followed by 3 × 100 µL PBS buffer rinsing steps to each flow channel using the solvent-exchange technique, as previously described [41]. The tBLM formation process was characterized using the tethaPod instrument (SDx Tethered Membranes) that produced a 25 mV alternating current (AC) signal with a frequency range of 0.1 Hz to 2000 Hz [90,91,92]. Data collection was performed using the tethaQUICK software program (SDx Tethered Membranes) and three independent measurements were performed per condition.

## 4. Conclusions

There is extensive interest in developing membrane-disrupting amphipathic peptides as pharmacological drug candidates to treat various types of cancer and microbial infections. Considering that lipid membranes are the main therapeutic target in such cases, there has long been an emphasis on applying biophysical measurement strategies to characterize the corresponding membrane-peptide interactions in order to gain mechanistic insight that can be useful for eventual clinical applications. Curiously, such biophysical strategies have been widely used to study antimicrobial and antiviral peptides, however, they have not been applied to investigate one of the most clinically advanced anticancer peptides, LTX-315, until the present study.

Building on past biological studies, the biophysical results presented herein establish that LTX-315 preferentially disrupts negatively charged lipid membranes and the extent of membrane disruption is greater at higher anionic lipid fractions. This enhanced membrane disruption supports that electrostatic interactions play a critical role in modulating membrane-disruptive activity. Interestingly, it was identified that membrane curvature is an additional factor contributing to initial peptide attachment as LTX-315 could weakly attach to zwitterionic and positively charged membranes pertaining to sub-100 nm lipid vesicles, but not to corresponding supported lipid bilayers. Furthermore, across three tested model membrane platforms, the LTX-315 peptide demonstrated more extensive disruption of negatively charged membranes with lipid compositions bearing some resemblance to those of cancer cell membrane surfaces in terms of exposed PS lipids. As LTX-315 is a first-in-class anticancer peptide immunotherapy that is being explored for a wide range of cancer therapy applications in ongoing human clinical trials, it is critical to establish a strong mechanistic understanding of how LTX-315 functions and our biophysical findings provide direct evidence that the peptide preferentially and irreversibly disrupts negatively charged membranes in a manner that makes LTX-315 an excellent candidate for further clinical translation.

## Data Availability

The data presented in this study are available upon reasonable request from the corresponding authors.

## Supplementary Materials

The following supporting information can be downloaded at: https://www.mdpi.com/article/10.3390/ijms231810558/s1.

## Abbreviations

ACP	Anticancer peptide
AMP	Antimicrobial peptide
CD	Circular dichroism
DLS	Dynamic light scattering
DOPC	1,2-dioleoyl-*sn*-glycero-3-phosphocholine
DOPS	1,2-dioleoyl-*sn*-glycero-3-phospho-L-serine
DOEPC	1,2-dioleoyl-*sn*-glycero-3-ethylphosphocholine
EIS	Electrochemical impedance spectroscopy
EPC	Ethylphosphatidylcholine
PC	Phosphatidylcholine
PS	Phosphatidylserine
QCM-D	Quartz crystal microbalance-dissipation
SLB	Supported lipid bilayer
tBLM	Tethered bilayer lipid membrane
