# Supplementary material for: Biophysical Characterization of LTX-315 Anticancer Peptide Interactions with Model Membrane Platforms: Effect of Membrane Surface Charge
Source: Int J Mol Sci. 2022 Sep 12;23(18):10558. doi: 10.3390/ijms231810558 (PMC9501188; doi:10.3390/ijms231810558)
Supplement: Supplementary file 1 [file ijms-23-10558-s001.zip › ijms-1900169-supplementary.pdf]

Supplementary Information

Biophysical Characterization of LTX-315 Anticancer Peptide Interactions with Model Membrane Platforms: Effect of Membrane Surface Charge

Dong Jun Koo [1,†], Tun Naw Sut [1,†], Sue Woon Tan [1], Bo Kyeong Yoon [2], Joshua A. Jackman [1,*]

[1] School of Chemical Engineering and Translational Nanobioscience Research Center, Sungkyunkwan University, Suwon 16419, Republic of Korea

[2] School of Healthcare and Biomedical Engineering, Chonnam National University, Yeosu 59626, Republic of Korea

[†] These authors contributed equally to this work.

* Correspondence: jjackman@skku.edu (J.A.J.)

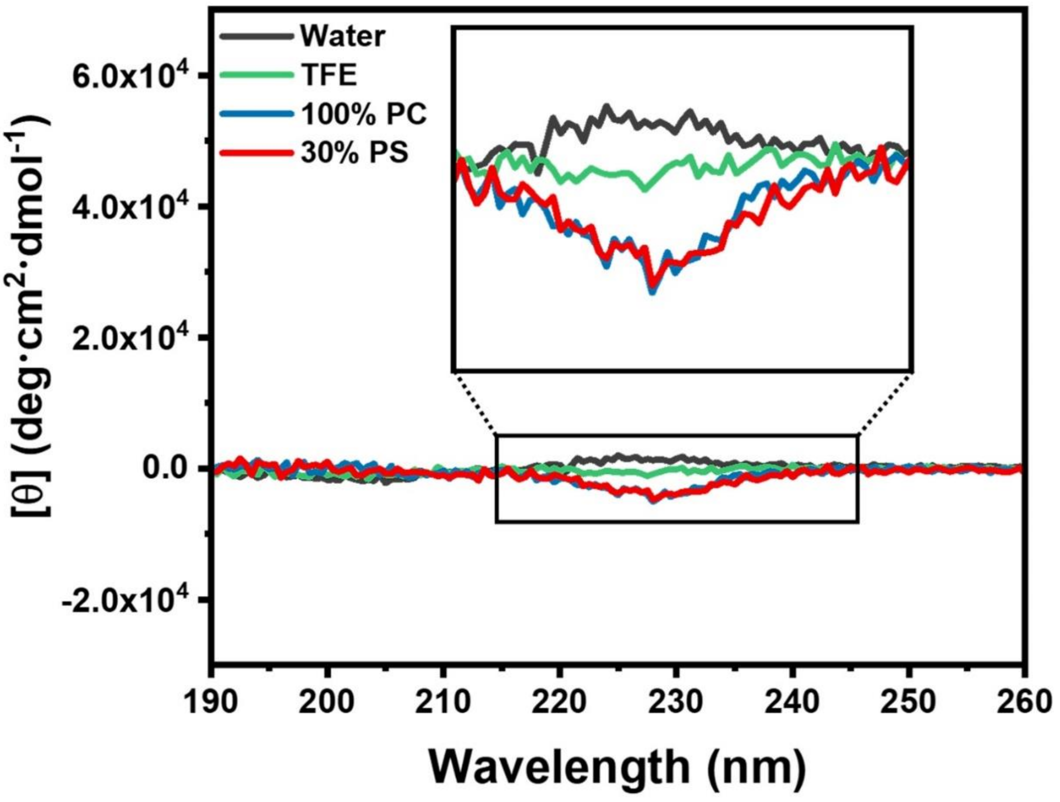

Figure S1. CD spectroscopy analysis of LTX-315 peptide. The measurements were conducted in aqueous (black line) and 50% v/v TFE (green line) conditions, and in the presence of 100 mol% DOPC lipid vesicles (blue line) or 70/30 mol% DOPC/DOPS lipid vesicles (red line). The peptide concentration was fixed at 100 μM. In the presence of vesicles, there was a slight increase in fractional helicity, but the changes were less than 15% and still negligible compared to typical cases of helix induction.

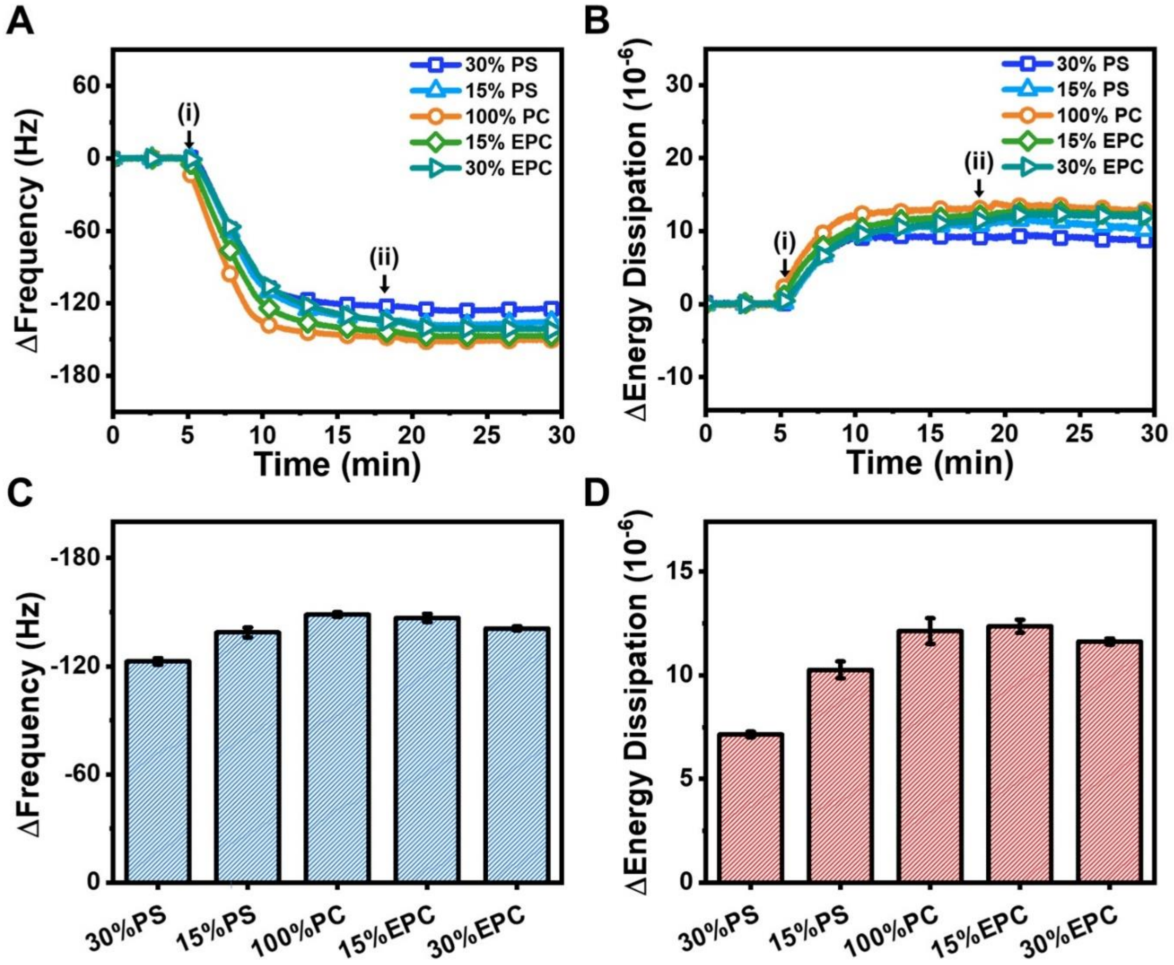

Figure S2. QCM-D measurements for tracking adsorption of vesicles with different lipid compositions onto titania surfaces. The QCM-D (**A**) resonance frequency and (**B**) energy dissipation shifts are presented as a function of time. After establishing measurement baselines in aqueous buffer, vesicles were added starting at t=5 min (arrow i) and buffer washing commenced from t=18 min (arrow ii). Summary of QCM-D (**C**) resonance frequency and (**D**) energy dissipation shifts corresponding to intact vesicle adlayers at saturation and data are reported as the mean ± standard deviation from n=3 measurements.

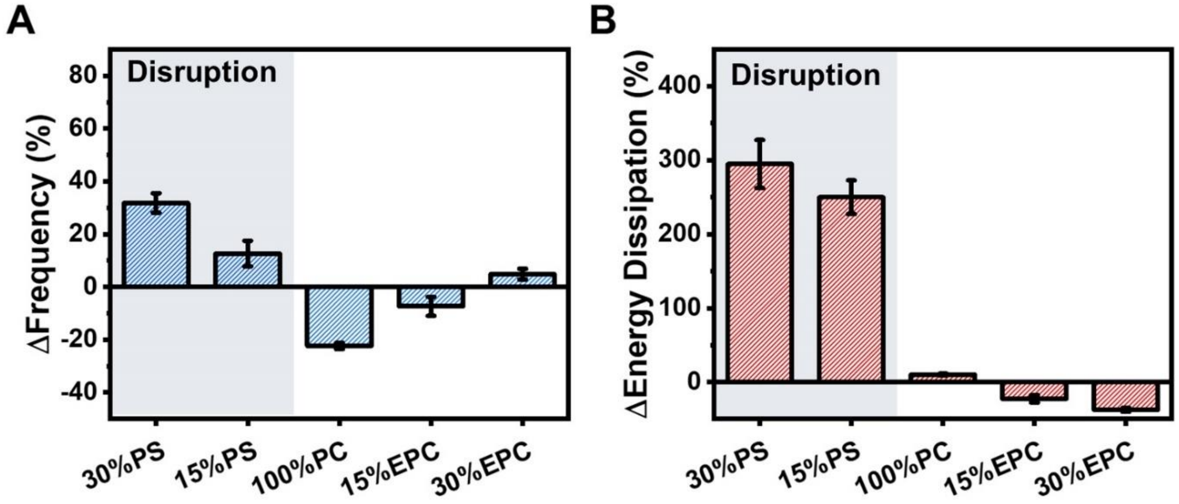

Figure S3. Summary of percentage changes in QCM-D measurement responses for LTX-315 peptide interactions with intact vesicle adlayers. The results correspond to the data in Figure 4 and are expressed in terms of the change in adlayer properties relative to the baseline values for the intact vesicle adlayers prior to peptide addition. The data are reported as the mean ± standard deviation from *n*=3 measurements.

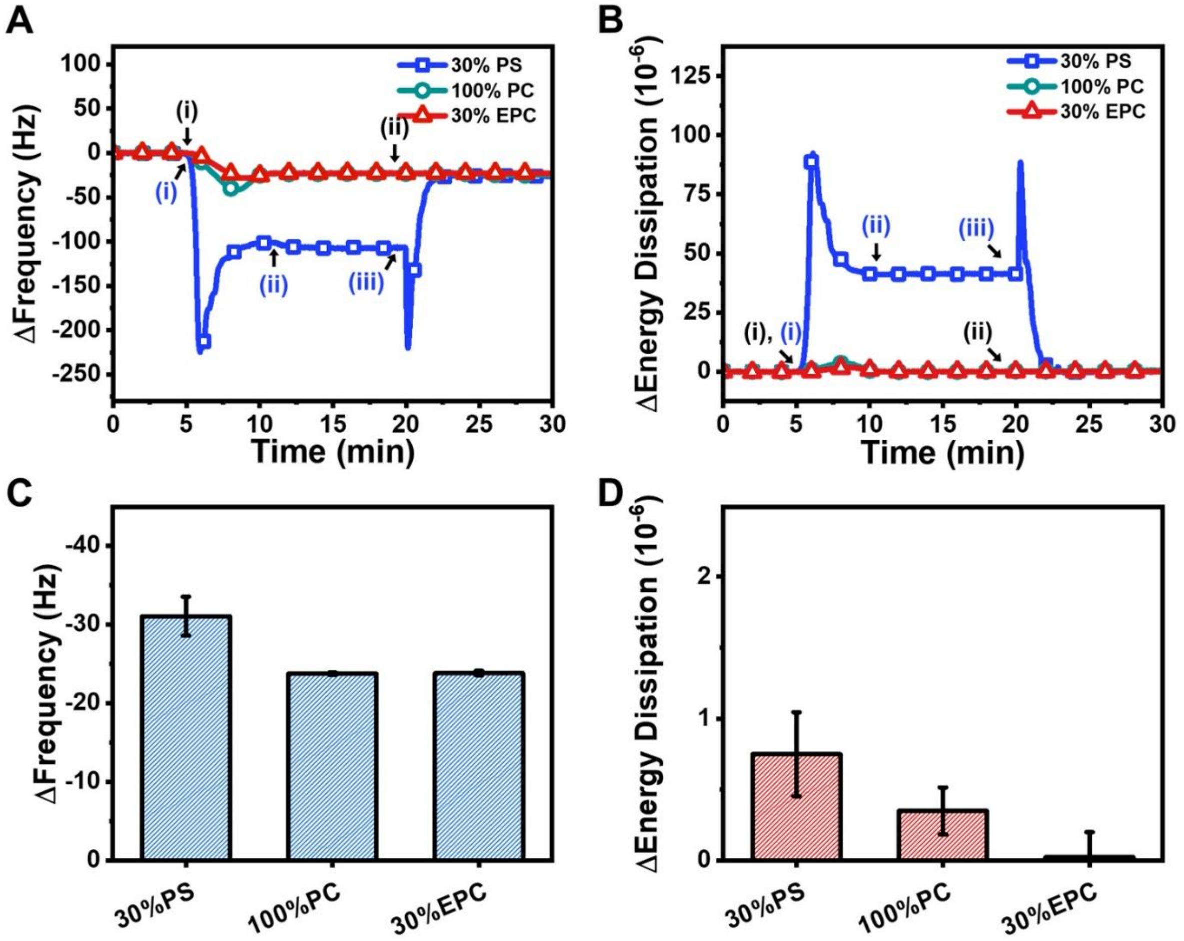

Figure S4. QCM-D measurements for tracking supported lipid bilayer formation with different lipid compositions on silica surfaces. The QCM-D (**A**) resonance frequency and (**B**) energy dissipation shifts are presented as a function of time. For 70/30 DOPC/DOPS lipid composition, the solvent-assisted lipid bilayer (SALB) method was used as follows: (1) measurement baselines were established in aqueous buffer, followed by (2) exchange to isopropanol at t=5 min (blue arrow i), (3) lipid addition at t=11 min (blue arrow ii), and (4) buffer washing at t=18 min (blue arrow iii). For 100 DOPC and 70/30 DOPC/DOEPC lipid compositions, the vesicle fusion method was used as follows: (1) measurement baselines were established in aqueous buffer, followed by (2) vesicle addition at t=5 min (black arrow i) and (3) buffer washing at t=18 min (black arrow ii). Summary of QCM-D (**C**) resonance frequency and (**D**) energy dissipation shifts corresponding to fabricated supported lipid bilayers and data are reported as the mean ± standard deviation from n=3 measurements.

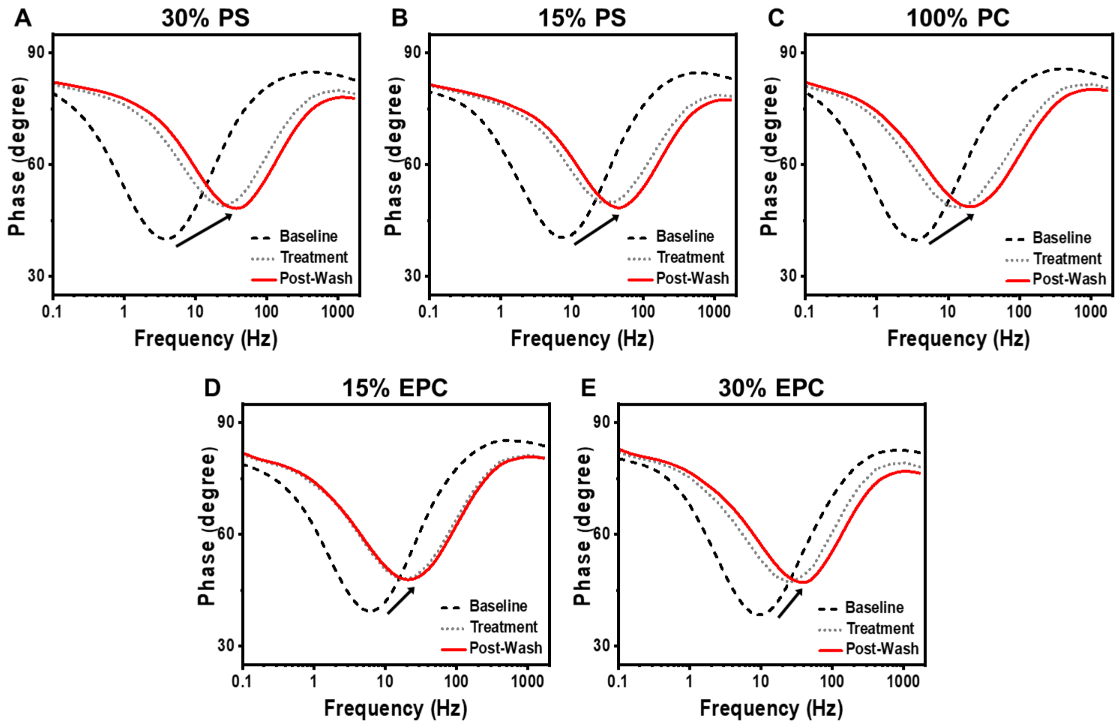

Figure S5. Bode plots of LTX-315 peptide interactions with tBLMs possessing different membrane surface charges. (**A**) Bode plots showing the shifts in phase values as a function of frequency for the 70/30 DOPC/DOPS tBLM platform in its original state (baseline), after addition of LTX-315 peptide (treatment) and after buffer rinsing (post-wash). Corresponding Bode plots for (**B**) 85/15 DOPC/DOPS, (**C**) 100 DOPC, (**D**) 85/15 DOPC/DOEPC, and (**E**) 70/30 DOPC/DOEPC tBLM platforms.
